# Cytosine–phosphate–guanine oligodeoxynucleotides alleviate radiation-induced kidney injury in cervical cancer by inhibiting DNA damage and oxidative stress through blockade of PARP1/XRCC1 axis

**DOI:** 10.1186/s12967-023-04548-y

**Published:** 2023-09-29

**Authors:** Deyu Zhang, Shitai Zhang, Zheng He, Ying Chen

**Affiliations:** 1 Department of Obstetrics and Gynecology, Shengjing Hospital of China Medical University, No.36 Sanhao Street, Shenyang, 110004 China; 2 Department of Nephrology, The First Hospital of China Medical University, No. 155 Nanjing Bei Street, Shenyang, 110001 Liaoning China

**Keywords:** Cervical cancer, Radiotherapy, Kidney injury, CpG-ODNs, PARP1, XRCC1, DNA damage, Oxidative stress

## Abstract

**Background:**

Radiotherapy can cause kidney injury in patients with cervical cancer. This study aims to investigate the possible molecular mechanisms by which CpG-ODNs (Cytosine phosphate guanine-oligodeoxynucleotides) regulate the PARP1 (poly (ADP-ribose) polymerase 1)/XRCC1 (X-ray repair cross-complementing 1) signaling axis and its impact on radiation kidney injury (RKI) in cervical cancer radiotherapy.

**Methods:**

The GSE90627 dataset related to cervical cancer RKI was obtained from the Gene Expression Omnibus (GEO) database. Bioinformatics databases and R software packages were used to analyze the target genes regulated by CpG-ODNs. A mouse model of RKI was established by subjecting C57BL/6JNifdc mice to X-ray irradiation. Serum blood urea nitrogen (BUN) and creatinine levels were measured using an automated biochemical analyzer. Renal tissue morphology was observed through HE staining, while TUNEL staining was performed to detect apoptosis in renal tubular cells. ELISA was conducted to measure levels of oxidative stress-related factors in mouse serum and cell supernatant. An in vitro cell model of RKI was established using X-ray irradiation on HK-2 cells for mechanism validation. RT-qPCR was performed to determine the relative expression of PARP1 mRNA. Cell proliferation activity was assessed using the CCK-8 assay, and Caspase 3 activity was measured in HK-2 cells. Immunofluorescence was used to determine γH2AX expression.

**Results:**

Bioinformatics analysis revealed that the downstream targets regulated by CpG-ODNs in cervical cancer RKI were primarily PARP1 and XRCC1. CpG-ODNs may alleviate RKI by inhibiting DNA damage and oxidative stress levels. This resulted in significantly decreased levels of BUN and creatinine in RKI mice, as well as reduced renal tubular and glomerular damage, lower apoptosis rate, decreased DNA damage index (8-OHdG), and increased levels of antioxidant factors associated with oxidative stress (SOD, CAT, GSH, GPx). Among the CpG-ODNs, CpG-ODN2006 had a more pronounced effect. CpG-ODNs mediated the inhibition of PARP1, thereby suppressing DNA damage and oxidative stress response in vitro in HK-2 cells. Additionally, PARP1 promoted the formation of the PARP1 and XRCC1 complex by recruiting XRCC1, which in turn facilitated DNA damage and oxidative stress response in renal tubular cells. Overexpression of either PARP1 or XRCC1 reversed the inhibitory effects of CpG-ODN2006 on DNA damage and oxidative stress in the HK-2 cell model and RKI mouse model.

**Conclusion:**

CpG-ODNs may mitigate cervical cancer RKI by blocking the activation of the PARP1/XRCC1 signaling axis, inhibiting DNA damage and oxidative stress response in renal tubule epithelial cells.

**Supplementary Information:**

The online version contains supplementary material available at 10.1186/s12967-023-04548-y.

## Background

Cervical cancer is a common gynecological cancer that contributes to cancer-related deaths throughout the world [1], and radiotherapy is a standard treatment strategy for this malignancy [2]. However, radiation used in radiotherapy can induce kidney toxicity, resulting in double-stranded breaks (DSBs) in the DNA and death of renal endothelial, tubular as well as glomerular cells [3]. Radiation can induce DNA damage and cell apoptosis in the kidneys [4]. In addition, radiation results in oxidative stress and prolongation of cell injury [5]. In this context, it is of significance to further the understanding of mechanism regarding DNA damage and oxidative stress in radiation-induced kidney injury (RKI) in cervical cancer.

Cytosine–phosphate–guanine oligodeoxynucleotides (CpG-ODNs) are artificial unmethylated CpG motifs originally discovered in bacterial DNA [6]. As previously reported, CpG-ODN could confer ameliorating effect against radiation-induced lung injury in a mouse model [7]. CpG-ODN also upregulated G-CSF and IL-6 to improve irradiation-induced injuries [8]. In addition, CpG-ODN prevented normal immune cells from gamma-irradiation-induced death [9]. It should be noted that the bioinformatics analysis conducted in the present study predicted that CpG-ODNs might regulate the downstream targets poly ADP-ribose) polymerase 1 (PARP1)/X-ray repair cross complementing 1 (XRCC1) in RKI in cervical cancer. PARP1 has been highlighted as an RNA-binding protein in multiple kidney diseases with tubular cell injury [10]. Suppression of PARP1 has been unfolded to exert protection against cisplatin-induced kidney injury [11, 12]. As previously reported, the application of novel PARP inhibitors has the potential of rescuing normal cells from oxidative damage during radiotherapy-induced ischemia–reperfusion injury [13]. Knockout of XRCC1 had a defect in repair of hydrogen peroxide and camptothecin-induced DNA damage, but this could be rescued by further deletion of PARP1 [14]. PARP1 can participate in cellular responses to oxidative stress by forming PAR for response to DNA damage, and XRCC1 can be recruited to sites of DNA damage dependent on PARP1 [15]. XRCC1 serving as a DNA-damage biomarker might participate in the regulation of cardiorenal syndrome-induced kidney injury [16]. The expression of PARP1 was found to be elevated by radiation in a dose-dependent manner [17]. Taking the aforementioned reports into consideration, this study proposed a hypothesis that CpG-ODNs might affect RKI in cervical cancer with the involvement of the regulation of the PARP1/XRCC1 axis, in hope of finding a novel direction for protection against RKI in cervical cancer.

## Materials and methods

### Ethical approval

The study was conducted under the approval of the Ethics Committee of China Medical University (KT2023763), and all procedures in the animal experiment were conducted in accordance with the *Guide for the Care and Use of Laboratory Animals*.

### Synthesis and purification of CpG-ODNs

Based on the available literature [7, 18], we used two types of CpG-ODNs for our study, CpG-ODN2216 and CpG-ODN2006 [19]. All CpG-ODNs were synthesized and purified by Sangon (Shanghai, China). CpG-ODNs were dissolved in DEPC water (1 mg/mL, Invitrogen, AM9906, Thermo Fisher Scientific, Rockford, IL) under sterile conditions, stored in a refrigerator at − 20 °C, and diluted with saline when used.

### Bioinformatics analysis

Relevant genes regulated by CpG-ODN (CpG-oligonucleotide) were obtained from the Comparative Toxicogenomics Database (CTD) (http://ctdbase.org) [20]. The cervical cancer-related microarray dataset GSE9750, comprising 33 tumor tissue samples and 24 normal cervical epithelial tissue samples, was obtained from the Gene Expression Omnibus (GEO) database (https://www.ncbi.nlm.nih.gov/gds) [21]. Differential analysis was performed using the "Limma" package in R software (version: 4.0), with the criteria of |log2(FoldChange)|> 2 and a significance level of *P* < 0.05 to select differentially expressed genes. The GeneCards database (https://www.genecards.org/) [22] was queried for the keyword "radiation nephropathy" to obtain relevant genes. The intersection of the above obtained genes from the CTD database for "CpG-ODNs," the analysis results of the cervical cancer-related microarray GSE9750 from the GEO database, and the relevant genes for "radiation nephropathy" obtained from the GeneCards database yielded the overlapping genes.

The protein–protein interaction network of the overlapping genes was obtained from the STRING database (https://string-db.org) [23]. Protein interaction analysis was performed using the "count" package in R software, and the top 10 nodes in the protein–protein interaction network were ranked based on Degree values (the number of connections between a node and other nodes, reflecting its centrality). The overlapping genes were subjected to Gene Ontology (GO) term analysis and KEGG enrichment analysis using the online analysis tool DAVID (http://david.ncifcrf.gov) [24].

The expression of PARP1 and HMGB1 in the TCGA-CESC (cervical cancer) dataset was analyzed using the Ualcan database (https://ualcan.path.uab.edu/analysis.html) [25]. The TCGA-CESC dataset consisted of 305 tumor tissue samples and 3 normal cervical epithelial tissue samples.

### 293T cells and HK-2 cells culture

HEK-293T cells were obtained from ATCC (CRL-3216, ATCC, USA). HK-2 cells, a human renal tubular epithelial cell line, were purchased from iCell Bioscience (iCell-h096, Shanghai, China). The cells were cultured in Dulbecco's modified Eagle's medium (DMEM) (11965092, Gibco, USA) supplemented with 10% fetal bovine serum (FBS) (10099141, Gibco, USA), 100 U/mL penicillin, and 100 μg/mL streptomycin (B540732, Sangon Biotech) at 37 °C in a humidified atmosphere with 5% CO_2_ [26, 27].

### Lentiviral vector construction

The lentiviral vectors carrying overexpression vector pCDH-CMV-MCS-EF1α-copGFP (CD511B-1, System Biosciences, Mountain View, CA) and shRNA vector pGreenPuro-CMV-shRNA-Lentivector (SI505A-1, System Biosciences) were purchased. Lentivirus-based PARP1 and XRCC1 overexpression or shRNA vectors were constructed, and the shRNA sequences are shown in Additional file 3: Table S1. Transfection of lentiviral vectors into 293T cells using Opti-MEM medium (31985070, Gibco, USA) and Lipofectamine 3000 reagent (L3000015, Invitrogen, New York, California, USA). After 20 h, the medium was replaced with 12 mL of medium supplemented with 5% fetal bovine serum (FBS). After 48 h of transduction, the virus-containing supernatant was collected, filtered through a 0.45 μm cellulose acetate filter (HAWG04700, MF-Millipore, Billerica, MA), and stored in a refrigerator at − 80 °C.

To construct human renal tubular epithelial cell line HK-2 (iCell-h096, Sebacon (Shanghai) Biotechnology Co., Ltd.) with PARP1/XRCC1 overexpression or shRNA vector, HK-2 cells at a density of 40% were incubated with virus-containing mix (MOI [Multiplicity of Infection] of 20) for 8 h. After 24 h, an additional 10 μg/mL puromycin was added to screen HK-2 and the culture was maintained for 4 weeks to construct stable-transduced cell lines.

### Construction of RKI mouse model

Eighty-eight 6–8 week old female C57BL/6JNifdc mice were purchased from Vitalriver; (Beijing, China). They were housed in single cages under constant temperature and humidity. After 1 week of acclimation, mice were used as control mice or subjected to modeling as RKI mice. RKI mice were irradiated with X-rays at a dose of 14 Gy, with a dose rate of 2.35 cGy/min at the kidney site, 5 days per week for 4 weeks (180 times of rotation during each irradiation to optimize dose uniformity) [28, 29]. After 4 weeks, a fully automated biochemical analyzer (AU5800, Beckman Coulter Inc., Chaska, MN) was used to measure serum blood urea nitrogen (BUN) and creatinine values. If the serum BUN and creatinine levels of the modeled mice were 2–3 times higher than those of the control mice, the modeling was considered successful and mice with successful modeling were selected for subsequent experiments. Control mice were not treated with X-ray irradiation.

A total of 24 control mice were not treated with X-ray irradiation, or intraperitoneally injected with ODN2006 or ODN2216 (n = 8). Meanwhile, 56 RKI model mice were not further treated, or intraperitoneally injected with ODN2006, ODN2216, saline + lentiviral vector carrying overexpression (oe)-negative control (NC), ODN2006 + lentiviral vector carrying oe-NC, ODN2006 + lentiviral vector carrying oe-PARP1, or ODN2006 + lentiviral vector carrying oe-XRCC1 (n = 8) (Additional file 3: Table S2).

At 1 week before X-ray irradiation, lentiviral injections (1 × 10^6^ TU/mouse) [30, 31] were performed by tail vein injection 1 h before CpG-ODNs injection, twice a week for 4 weeks. Mice were injected with 10 μg/mL CpG-ODNs or saline via intraperitoneal injection 5 times per week and 4 times per week thereafter. X-ray irradiation was used, 5 times per week for 4 weeks.

### Construction of an in vitro cell model of RKI

Human renal cortical proximal tubule epithelial cells HK-2 (iCell-h096, iCell Bioscience Inc., Shanghai, China) were cultured in HK-2 cell medium (iCell-h096-001b, iCell Bioscience Inc.).

HK-2 cells were grouped as follows:

1. Control cells (without any treatment), HK-2 cells treated with radiation (given X-ray irradiation treatment), and HK-2 cells treated with radiation + ODN2006 (given 5 μM ODN2006 treatment and X-ray irradiation treatment).
2. HK-2 cells were treated with short hairpin RNA (sh)-NC (lentiviral vector carrying sh-NC), sh-PARP1 (lentiviral vector carrying sh-PARP1-1 and sh-PARP1-2), ODN2006 + sh-PARP1 (transduced with lentiviral vector carrying sh-PARP1 and then treated with ODN2006), oe-NC + sh-NC (transduced with lentiviral vector carrying sh-NC and oe-NC), oe-NC + sh-XRCC1 (transduced with lentiviral vector carrying oe-NC and sh-XRCC1-1/sh-XRCC1-2), saline + oe-NC (transduced with lentiviral vector carrying oe-NC and then cultured with saline), ODN2006 + oe-NC (transduced with lentiviral vector carrying oe-NC and then treated with ODN2006), ODN2006 + oe-PARP1 (transduced with lentiviral vector carrying oe-PARP1 and then treated with ODN2006), ODN2006 + oe-XRCC1 (transduced with lentiviral vector carrying oe-XRCC1 and then treated with ODN2006), followed by X-ray irradiation treatment. Each mouse was intraperitoneally injected with 50 μg of CpG-ODNs or saline solution, five times per week, followed by every fourth session. The mice were exposed to X-ray irradiation, five times per week, for a total of 4 weeks [7, 8].

### RT-qPCR

Total RNA was extracted from tissues and cells using TRIzol (15596026, Thermo Fisher Scientific), and a nanodrop 2000 micro-UV spectrophotometer (Thermo Fisher Scientific) was utilized to detect the concentration and purity of the extracted total RNA. The RNA was reversely transcribed into cDNA according to the instructions of PrimeScript RT reagent Kit (RR047A, Takara, Shiga, Japan). The synthesized cDNA was subjected to RT-qPCR detection using Fast SYBR Green PCR Kit (Thermo Fisher Scientific), and three replicates were set up for each well. GAPDH was used as the internal reference. The 2^−ΔΔCt^ method was utilized to analyze the relative mRNA expression. The primer sequences used for RT-qPCR in this study are shown in Additional file 3: Table S3 [32].

### Co-IP assay

To determine the interaction of XRCC1 and PARP1, we co-transfected HK-2 cells with vectors expressing Myc-PARP1 and XRCC1-flag. For immunoprecipitation, we used the Universal Magnetic Co-IP kit (cat#54002, Active Motif, Carlsbad, CA) to extract proteins according to the manufacturer's instructions. Briefly, for co-IP of Myc-PARP1 and XRCC1-Flag proteins in cells with ectopic expression of Myc-PARP1 and XRCC1-Flag, we used anti-Myc (sc-40, Santa Cruz Biotechnology, Santa Cruz, CA) and anti-Flag (A2220, Sigma-Aldrich, St. Louis, MO) agarose beads to pull down Myc-PARP1 and XRCC-Flag from cell lysates, respectively. Co-IP of endogenous PARP1 and XRCC1 was performed using anti-XRCC1 (#sc-56254, 1:100, Santa Cruz Biotechnology) or anti-PARP1 (ab227244, 1:100, Abcam, Cambridge, UK) antibodies. Control mouse IgG (sc-2025, 1:100, Santa Cruz Biotechnology) was used as a NC. Next, 25 μL protein G magnetic beads were added and incubated at 4 °C for 1 h. The precipitate was collected by centrifugation at 1000 g. The samples were washed four times with Co-IP/wash buffer and eluted with reduced loading buffer (130 mM Tris pH 6.8, 4% SDS, 0.02% bromophenol blue, 20% glycerol, and 100 mM DTT), followed by detection of interaction between XRCC1 and PARP1 using Western blot [33].

### Western blot

RIPA lysate (P0013C, Beyotime, Shanghai, China) containing PMSF was used to extract the total protein from the tissues, which was incubated on ice for 30 min and centrifuged at 4 °C for 10 min at 8000 g, followed by extraction of the supernatant. The total protein concentration was measured by BCA kits (23227, Thermo Fisher Scientific). The protein was subjected to SDS-PAGE, and transferred to a PVDF membrane, followed by blockade with 5% skimmed milk powder at room temperature for 1 h. Then, the PVDF membrane was incubated at 4 °C overnight with diluted primary antibodies against PARP1 (1:1000, ab191217, Abcam. Cambridge, UK), XRCC1 (1:1000, ab134056, Abcam) and GAPDH (ab181602, 1:2500, Abcam) and with HRP-labeled secondary antibody goat anti-rabbit IgG H&L (HRP) (ab97051, 1:2000, Abcam) for 1 h.

The ECL fluorescence assay kit (abs920, Absin Bioscience Inc.) was used for development. The protein bands were photographed using the Bio-Rad Image Analysis System (Bio-Rad Laboratories, Hercules, CA). Grayscale quantification was performed on each group of bands in the Western blot images using Image J analysis software (Version: V1.8.0), in which GAPDH was the internal reference protein [34].

### Validation of intracellular colocalization of PARP1 and XRCC1 proteins

HK-2 cells were divided into two groups: control group transfected with negative control shRNA, and another group interfered with endogenous PARP1 using shRNA vectors. The cells were placed on a 35 mm cell culture dish, PBS washed three times. They were then fixed with 4% cold paraformaldehyde for 20 min, followed by PBS washing three times. Permeabilization was performed using 0.2% Triton X-100 for 10 min, followed by PBS washing three times. Serum blocking was carried out for 30 min, followed by PBS washing three times. Cells were incubated overnight at 4 °C in the primary antibodies PARP1 (1:500, ab191217, Abcam, Cambridge, UK) and XRCC1 (1:500, ab134056, Abcam, Cambridge, UK), followed by PBS washing three times. The cells were then incubated with secondary antibodies Alexa Fluor 555 and Alexa Fluor 488 (Cell Signaling Technology, Beverly, MA, USA) for 2 h at room temperature (protected from light), or at 37 °C for 1.5 h, followed by PBS washing three times. Finally, DAPI was used to stain the nuclei, and fluorescence images were captured directly. PBS was washed off with distilled water, and the samples were mounted with glycerol and immediately imaged using an Olympus FLUOVIEW FV1000 confocal laser scanning microscope (Olympus Corporation, Tokyo, Japan) [35].

### CCK-8 assay

The CCK-8 kit (CA1210, Beijing Solarbio, Beijing, China) was used for cell viability assay. HK-2 cells at logarithmic growth stage were seeded in a 96-well plate with 1 × 10^4^ cells per well and cultured for 24 h. After 48 h of transfection, 10 μL of CCK-8 reagent was added at 0 h, 24 h, 48 h and 72 h after transfection, followed by incubation at 37 °C for 3 h. The absorbance values of each well at 450 nm were measured on a microplate reader [36].

### Determining the optimal concentration of CpG-ODNs

Once the RKI model mice were successfully constructed, 40 mice were divided into 5 groups for intraperitoneal injection of CpG ODNs at different concentrations: 0, 10, 20, 50, and 100 μg per mouse, with injections administered five times per week for a total of 4 weeks. After the injections, a fully automated biochemical analyzer (AU5800, Beckman Coulter, Beckman Coulter Trading (China) Co. Ltd.) was used to measure serum blood urea nitrogen (BUN) and serum creatinine levels.

For the in vitro cellular model of radiation-induced kidney injury, HK-2 cells were irradiated with X-rays at a dose of 8 Gy for 6 h. After radiation, HK-2 cells were seeded at a density of 1 × 104 cells per well in a 96-well plate and treated with different concentrations of CpG-ODN2006 (0, 1, 3, 5, 10, and 15 μM). Cell viability was then assessed using a CCK-8 assay kit (CA1210, Solaibao Technology, Beijing, China). At 0, 24, 48, and 72 h of incubation, 10 μL of CCK-8 reagent was added to each well, followed by incubation at 37 °C for 1 h. The absorbance at a wavelength of 450 nm was measured using an enzyme-linked immunosorbent assay reader, and a cell growth curve was plotted. The absorbance values were directly proportional to the number of proliferating cells in the culture medium [37].

### Immunofluorescence

HK-2 cells were exposed to 8 Gy of X-irradiation and recovered after 6 h. HK-2 cells were cultured on μ-slide VI access slides (Ibidi, Martinsried, German) and fixed with 4% paraformaldehyde at 4 °C for 30 min, followed by treatment with 0.2% TritonX-100 for 10 min. anti-γH2AX (ab2893, 1:100, Abcam) was then diluted with 3% BSA and incubated. Cells were stained with Alexa Fluor 488 goat anti-rabbit IgG (#4412S, 1:2000, Cell Signaling Technology, Beverly, MA) for 1 h at 37 °C and then with 4′,6-diamidino-2-phenylindole (MBD0020, Sigma-Aldrich). Finally, immunofluorescence images were obtained with a fluorescence microscope (ECLIPSE E800, Nikon, Tokyo, Japan), and five fields of view were randomly acquired for each sample [36].

### Caspase activity assay

Caspase activity was determined via enzymatic method using the fluorescent peptide substrate DEVD. Cells were extracted with 1% Triton X-100 and the lysate was added to an enzymatic reaction containing 50 mM DEVD (201608-14-2, Beijing Solarbio). The AFC reaction was performed for 60 min. The fluorescence at excitation wavelength of 360 nm/emission wavelength of 530 nm was monitored by a GENios plate reader. A standard curve was constructed with free AFC in each measurement. Using the standard curve, the fluorescence readings for each enzymatic reaction were converted into released AFC. Caspase activity was expressed as nmol of AFC released per mg of protein in the cell lysate [34].

### Hematoxylin–eosin (HE) staining

Mouse kidney tissue sections were stained with HE staining kits (PT001, Shanghai Bogoo Biological Technology Co., Ltd., Shanghai, China). In brief, the sections were stained with hematoxylin for 10 min, differentiated with 1% hydrochloric acid alcohol for 30 s, stained with eosin for 1 min, and dehydrated with gradient alcohol (concentrations of 70%, 80%, 90%, 95%, and 100%, respectively) for 1 min each. After being cleared with xylene, the sections were sealed with neutral gum, and the morphological changes were finally photographed and observed under a light microscope (BX50; Olympus Corp., Tokyo, Japan) [29].

### TUNEL staining

TUNEL Apoptosis Detection Kit-DAB (abs50022, Absin Bioscience Inc., Shanghai, China) was used to detect apoptosis in kidney tissues. In short, kidney tissue sections were incubated in TdT/nucleotide complex for 1 h at room temperature, and then the nuclei were labeled with horseradish peroxidase and diaminobenzidine. Counterstaining was then performed with hematoxylin. Apoptotic cells were observed under a light microscope and photographed. Five fields of view per section were randomly selected for cell counting to detect apoptosis. The apoptosis rate (%) was expressed as the percentage of TUNEL-positive cells, and the apoptosis rate = the number of apoptotic TUNEL-positive cells/total cell count × 100% [38].

### Detection of oxidative stress levels

Malondialdehyde (MDA) test kits (ml092884, Shanghai Meilian Biotechnology Co., Ltd., Shanghai, China) were used to detect MDA levels in the mouse serum and HK-2 cells via the TBA method. The levels of DNA damage index (8-OHdG) and each oxidative stress related factor in serum and cells were detected using mouse 8-OHdG (CSB-E10527m, Cusabio), human catalase (CAT) (JL14741, Shanghai Jianglai Biotechnology, Shanghai, China), mouse CAT (CSB-E14190m, Cusabio, Houston, TX), human superoxide dismutase (SOD) (CSB-E17064h, Cusabio), mouse SOD (JL12237, Shanghai Jianglai Biotechnology), human glutathione (GSH) (ml063305, Shanghai Meilian Biotechnology Co., Ltd.), mouse GSH (ml063305, Shanghai Meilian Biotechnology Co., Ltd.), mouse GSH (ml063305, Shanghai Meilian Biotechnology Co., Ltd.), human glutathione peroxidase (GPx) (JL10355, Shanghai Jianglai Biotechnology), mouse GPx (JL49904, Shanghai Jianglai Biotechnology) kits [39, 40].

### Statistical analysis

All data were processed using SPSS 24.0 statistical software (IBM Corp. Armonk, NY). All data were subjected to normal distribution and homogeneous variance tests, and data conforming to normal distribution were expressed in the form of mean ± standard deviation. Independent sample t-test was used for comparison between two groups, and data between multiple groups were compared by one-way ANOVA, followed by Tukey post hoc test. Absorbance values at multiple time points were compared using two-way ANOVA. *P* < 0.05 indicated statistically significant difference.

## Results

### CpG-ODNs attenuated RKI by inhibiting DNA damage and oxidative stress levels

**Fig. 1:**
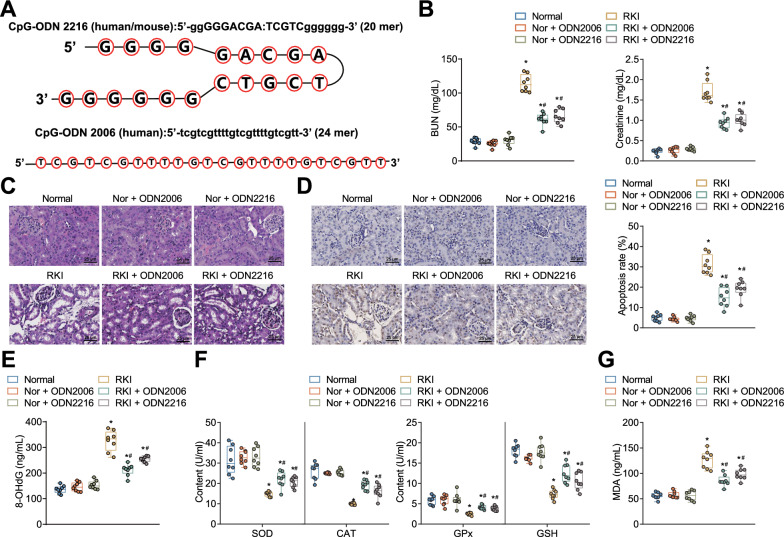
Protective effects of CpG-ODNs on RKI in relation to DNA damage and oxidative stress levels. **A** CpG-ODNs related sequence information; **B** Levels of blood urea nitrogen (BUN) and creatinine in mouse serum detected by automated biochemical analyzer; **C** Histopathological changes in mouse kidney tissues observed by HE staining (scale bar = 25 µm); **D** Levels of cellular apoptosis in mouse kidney tissues detected by TUNEL staining (scale bar = 25 µm), with brownish-gray indicating apoptotic cells; **E** Levels of DNA damage index (8-OHdG) in mouse serum detected by ELISA; **F** Levels of oxidative stress-related indexes in mouse serum detected by ELISA; **G** Levels of malondialdehyde (MDA) in mouse serum detected. * indicates *P* < 0.05 compared to the Normal group, # indicates *P* < 0.05 compared to the RKI group; each group consisted of 8 mice

First, we constructed an RKI mouse model through intraperitoneal injection of two CpG-ODNs solutions (CpG-ODN2006 and CpG-ODN2216) (Fig. 1a). To determine the optimal concentration of CpG-ODNs, we administered different concentrations of CpG-ODN2006 and CpG-ODN2216 to the RKI mice. The results showed that injecting 50 μg of either CpG-ODN2006 or CpG-ODN2216 significantly alleviated RKI in the mice (Additional file 1: Fig. S1). Therefore, we selected a concentration of 50 μg per mouse for the subsequent mouse experiments. The results of the kidney function analysis (Fig. 1b) demonstrated that, compared to the Normal group, the RKI group of mice exhibited significantly elevated levels of blood urea nitrogen (BUN) and creatinine. However, administration of the two CpG-ODNs treatments led to a significant reduction in BUN and creatinine levels in the RKI group of mice, whereas there were no significant changes in BUN and creatinine levels in the Normal group of mice following CpG-ODNs treatment.

The HE staining results (Fig. 1c) revealed that radiotherapy caused obvious kidney injury, mainly manifested by a large number of tubular epithelial cell necrosis, tubular dilatation, and glomerular hypertrophy; treatment with CpG-ODNs in the RKI mice significantly reduced tubular and glomerular injury, while no significant changes were observed in the kidney tissues of the control mice treated with CpG-ODNs. In addition, based on the TUNEL results (Fig. 1d), the apoptosis rate was significantly higher in the kidney tissues of the RKI mice compared with that in the control mice; treatment with CpG-ODNs significantly reduced the apoptosis rate in the kidney tissues of the RKI mice but had no significant change in that of the control mice.

We further examined the DNA damage index (8-OHdG) and oxidative stress levels in each group of mice. Our experimental results (Fig. 1e–g) revealed that compared with the control mice, the RKI mice showed a significant increase in 8-OHdG and a marked decrease in the production of oxidative stress-related factors SOD, CAT, GSH, GPx and a significant increase in MDA levels in kidney tissues; after the RKI mice were treated with CpG-ODNs, 8-OHdG was significantly diminished, accompanied by increases in SOD, CAT, GSH and GPx production and a notable decrease in MDA levels, while the control mice treated with CpG-ODNs showed no significant changes in the indexes.

The above results suggest that CpG-ODNs may attenuate RKI by suppressing DNA damage and oxidative stress levels.

### PARP1 might be a potential target for CpG-ODNs in RKI in cervical cancer

**Fig. 2:**
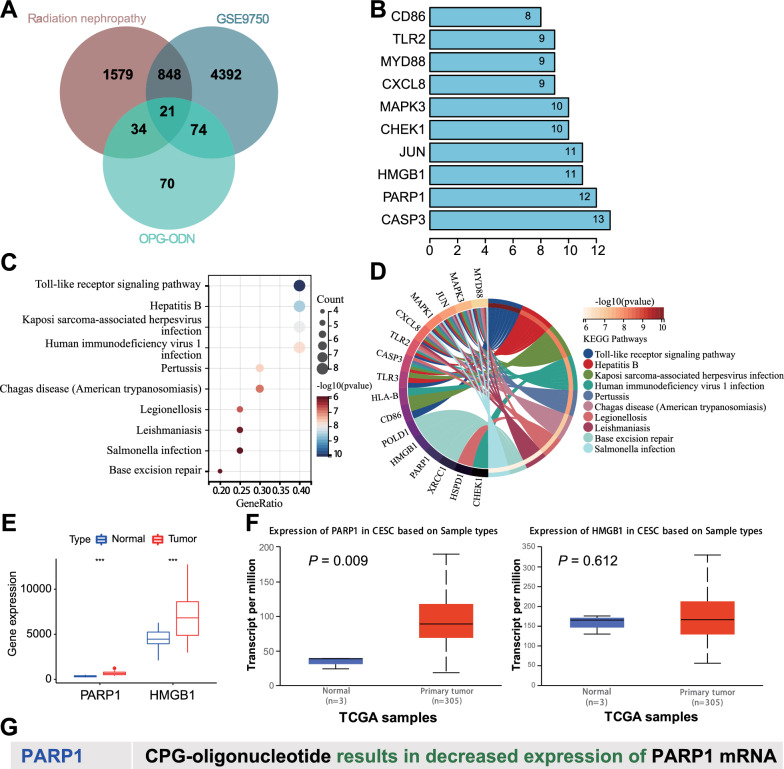
Bioinformatics analysis to screen potential targets of CpG-ODNs for treating RKI in cervical cancer. **A** Venn plot showing the intersection of genes related to "CpG-ODNs" obtained through the CTD database, DEGs in the cervical cancer-related microarray GSE9750 from the GEO database, and genes related to "radiation nephropathy" obtained from the GeneCards database. **B** STRING database analysis of 21 candidate intersection genes encoding the top 10 proteins with PPI Degree values. **C** Bubble plot of KEGG enrichment analysis of 21 candidate intersection genes. **D** Circle plot of KEGG enrichment analysis of 21 candidate intersection genes. **E** Differential expression data of PARP1 and HMGB1 in microarray GSE9750. Normal = 24, Tumor = 33. **F** Expression of PARP1 and HMGB1 in TCGA analyzed by Ualcan database. Normal = 3, Tumor = 305. **G** Regulation of PARP1 by CpG-ODNs in CTD database

In this study, we further explored the potential molecular mechanisms of CpG-ODNs for the treatment of RKI in cervical cancer. First, we searched the CTD database for 199 genes related to "CpG-ODNs". Then, we obtained a total of 5335 DEGs in the cervical cancer-related microarray GSE9750 from the GEO database, and further obtained 2482 genes related to "radiation nephropathy" using the GeneCards database. A total of 21 candidate genes were obtained by taking the intersection of the three databases (Fig. 2a).

The proteins encoded by the intersecting genes were further imported into the STRING database to obtain PPI relationships. The top 10 proteins were displayed according to Degree values, and the top three were CASP3, PARP1, and HMGB1 (Fig. 2b). In addition, we performed KEGG analysis of 21 candidate genes using the Sangerbox online tool and found that these 21 intersecting genes could be enriched in the entries such as Toll-like receptor signaling pathway, Base excision repair, etc. (Fig. 2c). It has been shown that ionizing radiation causes tissue damage by directly damaging DNA structures [29]. KEGG enrichment analysis showed that the genes on the Base excision repair entry were mainly XRCC1, PARP1, HMGB1, and POLD1, and intersection with the PPI results yielded intersection proteins PARP1 and HMGB1 (Fig. 2d).

Further differential analysis of the PARP1 and HMGB1 genes revealed that PARP1 showed high expression in both the cervical cancer chip GSE9750 and the TCGA-CESC dataset, while HMGB1 only exhibited high expression in the cervical cancer chip GSE9750 (Fig. 2e, f). Using the CTD database search, we found that CpG-ODNs mainly inhibited the expression of PARP1 (Fig. 2g).

Therefore, we speculate that PARP1 may be a potential target for CpG-ODNs in the treatment of RKI in cervical cancer, and CpG-ODNs may alleviate RKI in cervical cancer by downregulating PARP1 expression.

### CpG-ODNs inhibited DNA damage and oxidative stress in HK-2 cells through downregulation of PARP1 expression

**Fig. 3:**
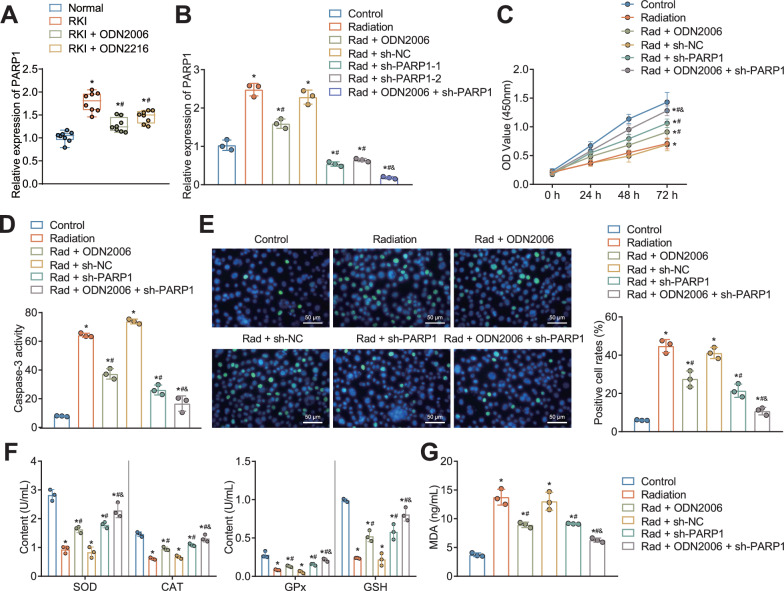
Effect of CpG-ODNs regulating PARP1 expression on in vitro RKI cell model. **A** RT-qPCR to detect the relative expression of PARP1 mRNA in the kidney tissue of RKI mice treated with CpG-ODN2006 or CpG-ODN2216. **B** RT-qPCR to detect the relative expression of PARP1 mRNA in radiation-treated HK-2 cells after CpG-ODN or sh-PARP1 treatment alone or in combination. **C** CCK-8 assay to detect the viability of radiation-treated HK-2 cells after CpG-ODN or sh-PARP1 treatment alone or in combination. **D** The Caspase activity in radiation-treated HK-2 cells after CpG-ODN or sh-PARP1 treatment alone or in combination as determined with enzymatic method. **E** Immunofluorescence assay to detect the γH2AX-positive cell ratio of radiation-treated HK-2 cells after CpG-ODN or sh-PARP1 treatment alone or in combination. Blue indicated DAPI fluorescence labeled nucleus, and green indicated labeled γH2AX. **F** Levels of oxidative stress-related factors (SOD, CAT, GSH, and GPx) in radiation-treated HK-2 cells after CpG-ODN or sh-PARP1 treatment alone or in combination. **G** MDA levels in radiation-treated HK-2 cells after CpG-ODN or sh-PARP1 treatment alone or in combination. * *P* < 0.05 versus control mice or control cell. # *P* < 0.05 versus RKI mice, radiation-treated HK-2 cells or radiation-treated HK-2 cells treated with sh-NC. & *P* < 0.05 versus radiation-treated HK-2 cells treated with CpG-ODN2006. All cell experiments were repeated 3 times. n = 8 mice for each treatment

In addition, to investigate whether CpG-ODNs attenuate RKI in cervical cancer by regulating PARP1, we examined the expression of PARP1 in the kidney tissues of RKI mice treated with two CpG-ODNs (CpG-ODN2006 and CpG-ODN2216). Our experimental results showed that PARP1 expression was significantly increased in the kidney tissues of the RKI mice compared with that in the control mice. Moreover, PARP1 expression was notably reduced in the RKI mice treated with CpG-ODNs, and CpG-ODN2006 with better treatment effect than CpG-ODN2216 was selected for the follow-up experiment (Fig. 3a).

We established an in vitro model of kidney injury cells by subjecting the human renal tubular epithelial cell line HK-2 to X-ray irradiation. To determine the optimal concentration of CpG-ODN2006, we treated the irradiated cells with a gradient of CpG-ODN2006 concentrations. The results indicated that 5uM CpG-ODN2006 significantly increased the activity of HK-2 cells after radiation exposure (Additional file 2: Fig. S2). Thus, we selected 5uM CpG-ODN2006 for further experiments.

The RT-qPCR results demonstrated a significant increase in PARP1 mRNA expression in HK-2 cells of the Radiation group compared to the Control group (Fig. 3b). After silencing PARP1 in HK-2 cells of the Radiation group, the RT-qPCR results showed a significant decrease in PARP1 expression (Fig. 3b). Among the PARP1 silencers, sh-PARP1-1 exhibited the most effective silencing, thus it was selected for subsequent experiments (sh-PARP1). Furthermore, the addition of CpG-ODN2006 to HK-2 cells of the Radiation group significantly reduced PARP1 expression, and silencing PARP1 enhanced the inhibitory effect of CpG-ODN2006 on PARP1 expression.

We assessed the cell viability of HK-2 cells under different conditions using the CCK-8 assay. The results demonstrated a significant decrease in HK-2 cell viability in the Radiation group compared to the Control group (Fig. 3c). However, the addition of CpG-ODN2006 or silencing PARP1 in the Radiation group resulted in a significant increase in HK-2 cell viability. The results from the Caspase activity assay revealed a significant increase in Caspase activity in HK-2 cells of the Radiation group compared to the Control group (Fig. 3d). However, the addition of CpG-ODN2006 or silencing PARP1 in the Radiation group led to a significant decrease in Caspase activity in HK-2 cells. Silencing PARP1 further enhanced the inhibitory effect of CpG-ODN2006 on Caspase activity in the Radiation group.

As shown by Fluorescence imaging and quantitative assay results of γH2AX (Fig. 3e), compared with that in the control cells, the ratio of γH2AX-positive cells was significantly higher in HK-2 cells treated with radiation. The ratio of γH2AX-positive cells in HK-2 cells treated with radiation was significantly lower after silencing of PARP1 or addition with CpG-ODN2006. The inhibitory effect on ratio of γH2AX positive cells in HK-2 cells treated with radiation was more pronounced upon combination of PARP1 silencing and CpG-ODN2006.

The results of the oxidative stress level assay in the in vitro RKI cell model (Fig. 3f, g) showed significantly decreased SOD, CAT, GSH, and GPx levels and increased MDA levels in the radiation-treated HK-2 cells. In addition, the addition of CpG-ODNs or silencing of PARP1 resulted in marked increases in the SOD, CAT, GSH, GPx levels and a decline in the MDA levels in the radiation-treated HK-2 cells, while these effects were more obvious upon combination of PARP1 silencing and CpG-ODN2006.

The above results suggest that CpG-ODNs can inhibit DNA damage and oxidative stress in HK-2 cells by downregulating PARP1 expression.

### PARP1 promoted DNA damage and oxidative stress in HK-2 cells by recruiting XRCC1 to form a complex

**Fig. 4:**
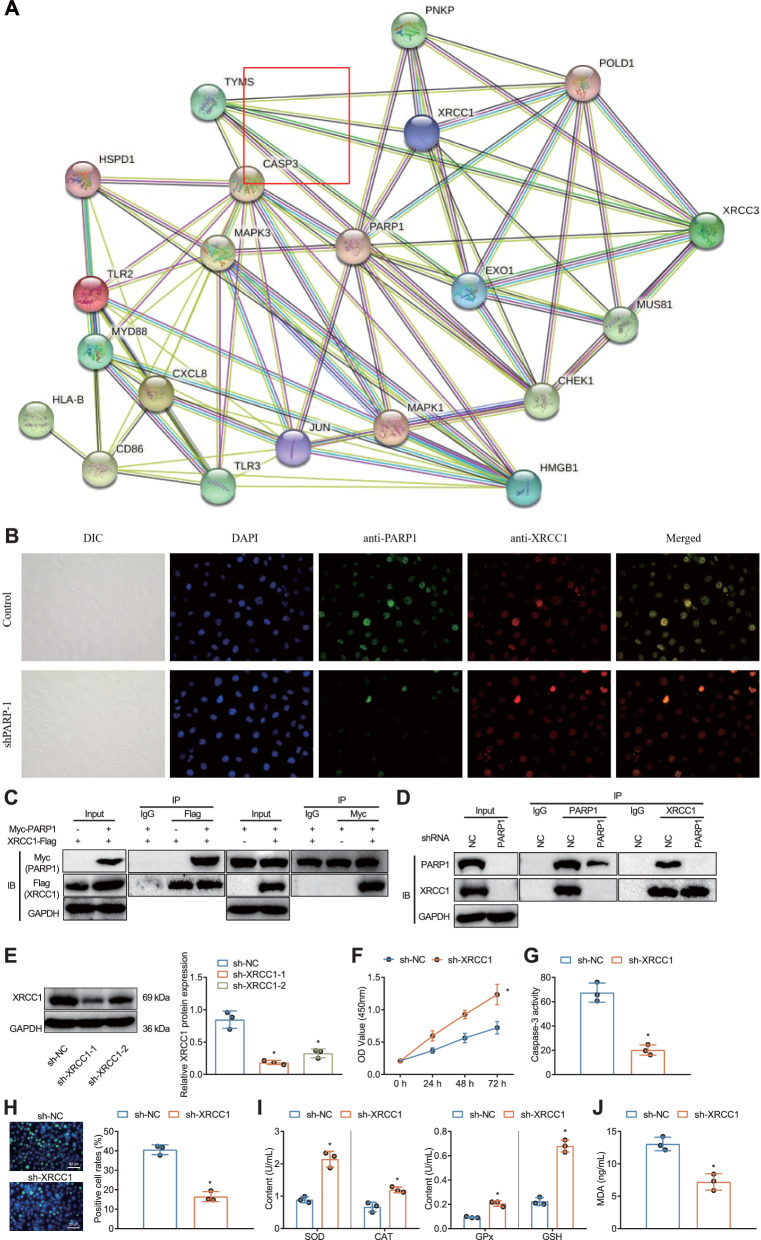
Effects of Interaction between PARP1 and XRCC1 on DNA Damage and Oxidative Stress Response in HK-2 Cells. **A** Interaction network of candidate target gene proteins obtained from the String database; **B** Immunofluorescence co-localization of PARP1 (green), XRCC1 (red), and DAPI (blue); **C**, **D** Co-IP validation of the interaction between PARP1 and XRCC1; **E** Western blot analysis of XRCC1 expression levels in different groups of HK-2 cells; **F** CCK-8 assay to measure cellular activity in different groups of HK-2 cells; **G** Enzymatic method to measure Caspase activity in different groups of HK-2 cells; **H** Immunofluorescence assay to assess the percentage of positive γH2AX cells, with blue DAPI fluorescence marking the cell nucleus and green marking γH2AX (scale bar = 50 µm); **I** Measurement of oxidative stress-related factors' anti-peroxidation levels in different groups of HK-2 cells; **J** Measurement of MDA levels in different groups of HK-2 cells. * denotes significant difference compared to the sh-NC group (*P* < 0.05). All cell experiments were repeated 3 times

Furthermore, we predicted the interaction between PARP1 and XRCC1 using the String database (Fig. 4a). Subsequently, we used immunofluorescence techniques to validate the intracellular localization of XRCC1 and PARP proteins in HK-2 cells. Confocal microscopy showed that XRCC1 co-localized with PARP1 protein (Fig. 4b). Additionally, KEGG enrichment analysis confirmed the participation of both PARP1 and XRCC1 in DNA base repair (Fig. 2e). It has been shown in the literature that PARP1 and XRCC1 can form a complex and XRCC1 appears to be involved in PARP1-mediated apoptosis [41–43].

To further explore whether PARP1 and XRCC1 have an interaction relationship in renal tubular cells, we first verified it using Co-IP assay, and the results showed that there was an interaction between exogenous XRCC1-Flag and Myc-PARP1 (Fig. 4c). We used shRNA vector to interfere with endogenous PARP1. In HK-2 cells overexpressing PARP1, PARP1 was able to interact with XRCC1, and after interfering with PARP1, their interaction was attenuated (Fig. 4d).

To further investigate the role of XRCC1 in RKI, we interfered XRCC1 in radiation-treated HK-2 cells. Western blot assay results (Fig. 4e) showed that XRCC1 protein expression was significantly reduced in HK-2 cells after treatment with sh-XRCC1, and sh-XRCC1-1 with optimal interference effect was selected for subsequent experiments.

We further explored the effect of XRCC1 on the RKI cell model. The results (Fig. 4f–j) showed that silencing of XRCC1 resulted in a significant increase in cell viability, a marked decrease in Caspase activity, a decrease in the proportion of γH2AX-positive cells, a significant increase in the production of oxidative stress-related factors SOD, CAT, GSH, and GPx, and a decrease in MDA levels in radiation-treated HK-2 cells.

The above results suggest that PARP1 may recruit XRCC1 to form a complex, thereby promoting DNA damage and oxidative stress response in renal tubular cells.

### CpG-ODNs inhibited DNA damage and oxidative stress in HK-2 cells by blocking PARP1/XRCC1 axis activation

**Fig. 5:**
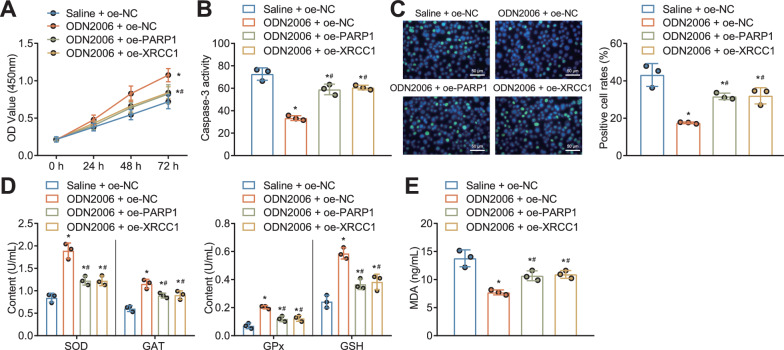
Effect of CpG-ODNs on DNA damage and oxidative stress in HK-2 cells by regulating PARP1/XRCC1 axis. **A** CCK-8 assay for detection of viability of radiation-treated HK-2 cells in response to CpG-ODN2006 alone or combined with oe-PARP1 or oe-XRCC1. **B** Caspase activity in radiation-treated HK-2 cells in response to CpG-ODN2006 alone or combined with oe-PARP1 or oe-XRCC1 as determined with enzymatic method. **C** Immunofluorescence assay to detect the γH2AX-positive cell ratio of radiation-treated HK-2 cells in response to CpG-ODN2006 alone or combined with oe-PARP1 or oe-XRCC1. Blue indicated DAPI fluorescence labeled nucleus, and green indicated labeled γH2AX. **D** ELISA detection of levels of oxidative stress-related factors (SOD, CAT, GSH, GPx) in radiation-treated HK-2 cells in response to CpG-ODN2006 alone or combined with oe-PARP1 or oe-XRCC1. **E** ELISA detection of MDA levels in radiation-treated HK-2 cells in response to CpG-ODN2006 alone or combined with oe-PARP1 or oe-XRCC1. * *P* < 0.05 versus HK-2 cells treated with saline + oe-NC. # *P* < 0.05 versus HK-2 cells treated with CpG-ODN2006 + oe-NC. All cell experiments were repeated three times

Next, we further explored the effects of CpG-ODNs regulating PARP1/XRCC1 axis on DNA damage and oxidative stress in HK-2 cells. Our experimental results demonstrated that overexpression of either PARP1 or XRCC1 reversed the inhibitory effect of CpG-ODN2006 on PARP1 or XRCC1 in HK-2 cells after radiation. In addition, we also found that overexpression of PARP1 or XRCC1 reversed the inhibitory effects of CpG-ODN2006 on DNA damage and oxidative stress in HK-2 cells after radiation (Fig. 5a–e).

The aforementioned results suggest that CpG-ODNs can inhibit DNA damage and oxidative stress response in HK-2 cells via inactivation of PARP1/XRCC1 axis.

### CpG-ODNs attenuated RKI by inhibiting DNA damage and oxidative stress

**Fig. 6:**
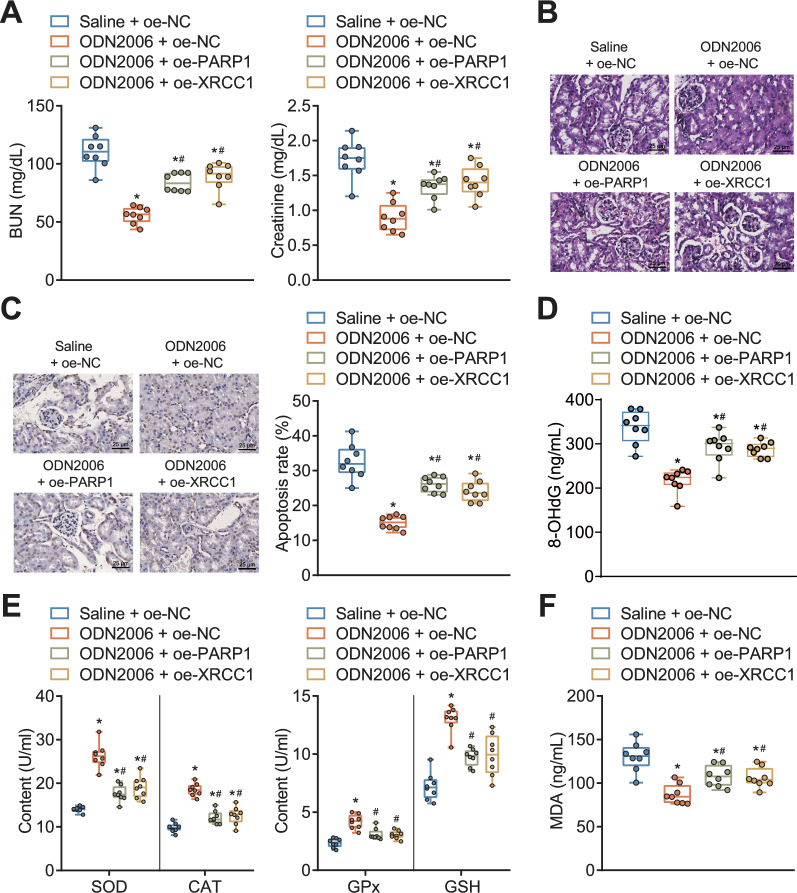
Effect of CpG-ODNs regulating PARP1/XRCC1 signaling axis on RKI mice. **A** Serum BUN and creatinine levels in RKI mice in response to CpG-ODN2006 alone or combined with oe-PARP1 or oe-XRCC1 detected by an automatic biochemical analyzer. **B** HE staining to observe the pathological changes in the kidney tissue in RKI mice in response to CpG-ODN2006 alone or combined with oe-PARP1 or oe-XRCC1. **C** TUNEL to detect the level of apoptosis in the kidney tissue in RKI mice in response to CpG-ODN2006 alone or combined with oe-PARP1 or oe-XRCC1. Brown-grey color represents apoptotic cells. **D** ELISA detection of DNA damage index (8-OHdG) in serum of RKI mice in response to CpG-ODN2006 alone or combined with oe-PARP1 or oe-XRCC1. **E** ELISA detection of levels of oxidative stress-related factors (SOD, CAT, GSH, and GPx) in serum of RKI mice in response to CpG-ODN2006 alone or combined with oe-PARP1 or oe-XRCC1. **F** ELISA detection of MDA levels in serum of RKI mice in response to CpG-ODN2006 alone or combined with oe-PARP1 or oe-XRCC1. * *P* < 0.05 versus RKI mice treated with saline + oe-NC. # *P* < 0.05 versus RKI mice treated with ODN2006 + oe-NC. n = 8 mice for each treatment

We further explored the effect of CpG-ODNs regulating PARP1/XRCC1 axis on RKI through in vivo animal experiments. Our experimental results (Fig. 6a–f) showed that overexpression of either PARP1 or XRCC1 reversed the protective effects of CpG-ODN2006 on RKI mice, increased the effects of serum BUN and creatinine levels, and aggravated tubular and glomerular injury and cell apoptosis.

Taken together, CpG-ODNs can inactivate PARP1/XRCC1 axis, inhibiting DNA damage and oxidative stress response, which attenuates RKI.

## Discussion

RKI occurs during radiotherapy, with elusive pathophysiology [44]. Herein, we set out to investigate the role of CpG-ODNs in RKI in cervical cancer and our results found that CpG-ODNs could inactivate the PARP1/XRCC1 axis to prevent RKI in cervical cancer.

Results of the study revealed that CpG-ODNs attenuated RKI by inhibiting DNA damage and oxidative stress levels. CpG-ODN played an alleviatory role in radiation-induced lung injury in a mouse model [7]. Several studies have suggested CpG-ODNs as an important inhibitor for radiation-induced pulmonary fibrosis [45, 46] and bone marrow hemopoiesis injury [47]. CpG-ODNs can provide protection against irradiation possibly through DNA damage repair and apoptosis inhibition [48]. CpG-ODNs can regulate oxidative stress in cancer patients [49]. CpG ODNs yielded preventive effects on acute radiation-induced lung injury partially by inhibiting the injury of reactive oxygen species and oxidative stress [50]. Nevertheless, the function of CpG-ODNs in RKI through regulation of DNA damage and oxidative stress has been rarely reported.

Subsequently, we found that CpG-ODNs inhibited DNA damage and oxidative stress in HK-2 cells through downregulation of PARP1 expression. Downregulated PARP1 was revealed to protect against irradiation-induced organ injury such as brain injury [51] and premature ovarian failure [52]. Moreover, inhibition of PARP1 degradation due to insulin-like growth factor binding protein 7 could promote the development of acute kidney injury [53]. In our study, we unfolded that silencing of PARP1 further augmented the inhibitory effect of CpG-ODN2006 on the caspase activity, ratio of γH2AX positive cells in HK-2 cells in response to radiation, while resulting in marked increases in the SOD, CAT, GSH, GPx levels and a decline in the MDA levels. Of note, an increasing number of studies have reported the participation of PARP1 in the process of kidney injury through underlying regulation on DNA damage and oxidative stress. Doxorubicin induced DNA damage and oxidative stress in HK-2 cells via a PARP1-dependent way [54]. The decline in PARP1 expression by nicotinamide could suppress cisplatin-induced apoptosis in renal proximal tubular cells as well as acute kidney injury in mice, which might involve the diminished induction of γ-H2AX, a reflection of DNA damage response [11]. DNA damage resulted in PARP1 activation, and PARP1 inhibitor protected against cisplatin-induced nephrotoxicity [55]. Besides, it was unveiled that deficiency in PARP1 contributed to suppressed dysfunction of cisplatin-induced kidney dysfunction together with diminished oxidative stress and tubular necrosis, indicating the responsibility of PARP1 activation for cisplatin nephrotoxicity [56]. Another study showed that suppression of PARP1, a gene overactivated by oxidative DNA damage in multiple pathological conditions, brought about amelioration of the cisplatin-induced renal function impairment, as evidenced by alterations in serum BUN and creatinine levels and oxidative stress levels [12]. However, these previous studies, in the exploration of the role of PARP1, do not put focus on the kidney injury caused by radiotherapy. In our study, it is concluded that the function of CpG-ODNs in RKI is achieved through regulation of PARP1-induced DNA damage and oxidative stress.

Mechanistically, we further demonstrated PARP1 promoted DNA damage and oxidative stress in HK-2 cells by recruiting XRCC1 to form a complex. XRCC1 recruitment is promoted by PARP1, an enzyme that is activated following DNA damage, and even low levels of PARP1-synthesized ADP-ribosylation are sufficient for XRCC1 recruitment after oxidative stress [57]. XRCC1 can be recruited to DNA damage by interacting with its central BRCT domain with PAR chains produced by PARP1 [58]. Intriguingly, XRCC1, as an indicator of DNA damage, was highly expressed in chronic kidney disease [59]. Cellular expression of XRCC1 might be involved in the development of cardiorenal syndrome-induced kidney injury [16]. Elevated expression of PARP1 was observed in response to radiation in a dose-dependent manner [17]. DNA damage in advanced cortical cataracts remained at 72 h post-irradiation due to the presence of 8-OHG and a DNA repair protein, XRCC1 [60]. In our study, it is demonstrated that the PARP1/XRCC1 axis could regulate DNA damage and oxidative stress response in RKI.

This study provides new theoretical basis and molecular targets for the diagnosis and treatment of RKI. Due to the absence of unified and accurate medication guidelines for cervical cancer radiotherapy-induced kidney injury, a positive control group could not be established, resulting in insufficient evidence. Furthermore, limitations arise from financial and experimental constraints, leading to the use of only one HK2 cell line to establish the kidney injury model, as well as the unavailability of samples from RKI patients with cervical cancer or other cancers for analysis, thus limiting the generalizability of our findings. Additionally, the differential gene analysis data in this study were sourced from public databases, suggesting that subsequent studies incorporating transcriptome sequencing may yield more representative results. We acknowledge these shortcomings and plan to conduct further research in the subsequent stages to provide solutions for the treatment of cervical cancer radiotherapy-induced kidney injury.

Previous studies have reported that KSK CpG-ODN (K-CpG) effectively protects mice from radiation-induced lung injury by modulating innate immune responses [7]. Furthermore, research has indicated that all three types of ODNs can protect mice from radiation-induced damage, with B-grade ODNs demonstrating the most effective characteristics in radioprotection, possibly achieved through upregulating the expression of G-CSF and IL-6 [8]. In comparison to these studies, our findings suggest that CpG-ODNs may alleviate renal injury caused by cervical cancer radiotherapy (RKI) by inhibiting the activation of the PARP1/XRCC1 signaling axis, thus suppressing DNA damage and oxidative stress responses in renal tubular epithelial cells. Collectively, these studies highlight the significant role of CpG-ODNs in combating radiation injury. In our subsequent research, we will focus on elucidating the mechanisms of action of CpG-ODNs in more complex models of radiation damage and exploring more effective utilization of CpG-ODNs in treating radiation-induced bodily harm. Additionally, future investigations could translate the findings of this study into clinical practice by conducting clinical trials to verify the effectiveness and safety of CpG-ODNs or other PARP1/XRCC1 signaling axis inhibitors in treating RKI. Furthermore, personalized therapeutic strategies can be explored to optimize RKI treatment based on patients' genetic backgrounds and disease characteristics. The achievements of this series of studies are expected to make significant contributions to advancements in the field of radiation injury, offering new hope in improving patients' quality of life and enhancing the effectiveness of radiation therapy.

## Conclusions

**Fig. 7:**
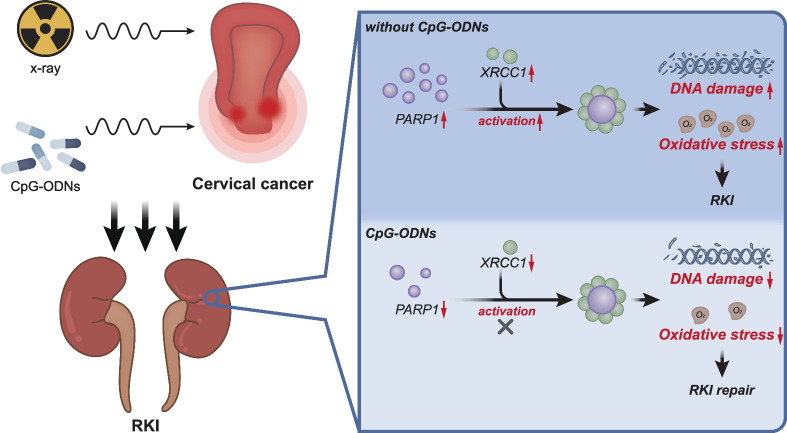
Schematic diagram of the molecular mechanism by which CpG-ODNs regulate PARP1/XRCC1 axis to affect DNA damage and oxidative stress response in RKI in cervical cancer. CpG-ODNs may attenuate RKI in cervical cancer by blocking PARP1/XRCC1 axis activation and inhibiting DNA damage and oxidative stress in renal tubular epithelial cells

To conclude, the present study provide evidence indicating that CpG-ODNs may attenuate RKI in cervical cancer by blocking PARP1/XRCC1 axis activation and inhibiting DNA damage and oxidative stress in renal tubular epithelial cells (Fig. 7). This finding provides a new mechanistic insight on RKI in cervical cancer. However, this study is only performed in cell and mouse models, with a lack of research on cervical cancer and other cancer related RKI patient samples. In addition, the differential analysis data in this study are sourced from public databases which requires further transcriptome sequencing analysis.

### Supplementary Information

**Additional file 1: Fig. S1.** Measurement of BUN and Creatinine Levels in Mouse Serum under Different Concentrations of CpG-ODNs Treatment Using an Automated Biochemical Analyzer. *Note*: Intraperitoneal injection in mice was used to validate the optimal concentration of ODN2006 and ODN2216. BUN and Creatinine levels in serum were measured at concentrations ranging from 0 to 100 μg. Significant differences were observed at concentrations of 50 μg and 100 μg.

**Additional file 2: Fig. S2.** Measurement of HK-2 cell viability under different concentrations of CpG-ODNs treatment and radiation-induced damage using CCK-8 assay.

**Additional file 3: Table S1.** shRNA sequences. **Table S2.** The treatment conditions of the different groups of mice. **Table S3.** Primer sequences for RT-qPCR.

## Data Availability

The datasets generated and/or analysed during the current study are available in the manuscript and Additional files 1–3.

## Abbreviations

DSBs: Double-stranded breaks
RKI: Radiation-induced kidney injury
CpG-ODNs: Cytosine–phosphate–guanine oligodeoxynucleotides
PARP1: Polymerase 1
XRCC1: Ray repair cross complementing 1
GEO: Gene Expression Omnibus
BP: Biological process
GO: Gene Onotology
PPI: Protein–protein interaction
FBS: Fetal bovine serum
BUN: Blood urea nitrogen
oe: Overexpression
NC: Negative control
sh: Short hairpin
HE: Hematoxylin–eosin
MDA: Malondialdehyde
CAT: Human catalase
SOD: Human superoxide dismutase
GSH: Human glutathione
GPx: Human glutathione peroxidase
CTD: Comparative toxicogenomics database
DEGs: Differentially expressed genes
MOI: Multiplicity of infection

## References

[1] Chen YF, Shen MR (2021). The important role of ion transport system in cervical cancer. Int J Mol Sci.

[2] Rodin D, Burger EA, Atun R, Barton M, Gospodarowicz M, Grover S (2019). Scale-up of radiotherapy for cervical cancer in the era of human papillomavirus vaccination in low-income and middle-income countries: a model-based analysis of need and economic impact. Lancet Oncol.

[3] Klaus R, Niyazi M, Lange-Sperandio B (2021). Radiation-induced kidney toxicity: molecular and cellular pathogenesis. Radiat Oncol.

[4] Xu D, Li H, Katsube T, Huang G, Liu J, Wang B (2022). Effects of concurrent exposure to chronic restraint-induced stress and total-body iron ion radiation on induction of kidney injury in mice. Int J Mol Sci.

[5] Azzam EI, Jay-Gerin JP, Pain D (2012). Ionizing radiation-induced metabolic oxidative stress and prolonged cell injury. Cancer Lett.

[6] Zhang Z, Kuo JC, Yao S, Zhang C, Khan H, Lee RJ (2021). CpG oligodeoxynucleotides for anticancer monotherapy from preclinical stages to clinical trials. Pharmaceutics.

[7] Park K, Dhupal M, Kim CS, Jung SH, Choi D, Qi XF (2020). Ameliorating effect of CpG-ODN (oligodeoxynucleotide) against radiation-induced lung injury in mice. Radiat Environ Biophys.

[8] Zhang P, Dong S, Guo J, Yang Y, Liu C, Li B (2017). CpG-oligodeoxynucleotides improved irradiation-induced injuries by G-CSF and IL-6 up-regulation. Cell Physiol Biochem.

[9] Sohn WJ, Lee KW, Choi SY, Chung E, Lee Y, Kim TY (2006). CpG-oligodeoxynucleotide protects immune cells from gamma-irradiation-induced cell death. Mol Immunol.

[10] Ke J, Liu F, Tu Y, Cai Z, Luo Y, Wu X (2021). PARP1-RNA interaction analysis: PARP1 regulates the expression of extracellular matrix-related genes in HK-2 renal proximal tubular epithelial cells. FEBS Lett.

[11] Wu W, Fu Y, Liu Z, Shu S, Wang Y, Tang C (2021). NAM protects against cisplatin-induced acute kidney injury by suppressing the PARP1/p53 pathway. Toxicol Appl Pharmacol.

[12] Mukhopadhyay P, Horvath B, Kechrid M, Tanchian G, Rajesh M, Naura AS (2011). Poly(ADP-ribose) polymerase-1 is a key mediator of cisplatin-induced kidney inflammation and injury. Free Radic Biol Med.

[13] Singh N, Pay SL, Bhandare SB, Arimpur U, Motea EA (2020). Therapeutic strategies and biomarkers to modulate PARP activity for targeted cancer therapy. Cancers (Basel).

[14] Reber JM, Bozic-Petkovic J, Lippmann M, Mazzardo M, Dilger A, Warmers R (2022). PARP1 and XRCC1 exhibit a reciprocal relationship in genotoxic stress response. Cell Biol Toxicol.

[15] Buelow B, Uzunparmak B, Paddock M, Scharenberg AM (2009). Structure/function analysis of PARP-1 in oxidative and nitrosative stress-induced monomeric ADPR formation. PLoS ONE.

[16] Yang CC, Chen YT, Wallace CG, Chen KH, Cheng BC, Sung PH (2019). Early administration of empagliflozin preserved heart function in cardiorenal syndrome in rat. Biomed Pharmacother.

[17] Tariq MA, Soedipe A, Ramesh G, Wu H, Zhang Y, Shishodia S (2011). The effect of acute dose charge particle radiation on expression of DNA repair genes in mice. Mol Cell Biochem.

[18] Lebre MC, van der Aar AM, van Baarsen L, van Capel TM, Schuitemaker JH, Kapsenberg ML (2007). Human keratinocytes express functional Toll-like receptor 3, 4, 5, and 9. J Invest Dermatol.

[19] Krieg AM, Yi AK, Matson S, Waldschmidt TJ, Bishop GA, Teasdale R (1995). CpG motifs in bacterial DNA trigger direct B-cell activation. Nature.

[20] Davis AP, Wiegers TC, Johnson RJ, Sciaky D, Wiegers J, Mattingly CJ (2023). Comparative toxicogenomics database (CTD): update 2023. Nucl Acids Res.

[21] Barrett T, Wilhite SE, Ledoux P, Evangelista C, Kim IF, Tomashevsky M (2013). NCBI GEO: archive for functional genomics data sets-update. Nucl Acids Res.

[22] Stelzer G, Rosen N, Plaschkes I, Zimmerman S, Twik M, Fishilevich S (2016). The genecards suite: from gene data mining to disease genome sequence analyses. Curr Protoc Bioinformatics.

[23] Szklarczyk D, Kirsch R, Koutrouli M, Nastou K, Mehryary F, Hachilif R (2023). The STRING database in 2023: protein-protein association networks and functional enrichment analyses for any sequenced genome of interest. Nucl Acids Res.

[24] Sherman BT, Hao M, Qiu J, Jiao X, Baseler MW, Lane HC (2022). DAVID: a web server for functional enrichment analysis and functional annotation of gene lists (2021 update). Nucl Acids Res.

[25] Chandrashekar DS, Karthikeyan SK, Korla PK, Patel H, Shovon AR, Athar M (2022). UALCAN: an update to the integrated cancer data analysis platform. Neoplasia.

[26] Mao Y, Yan R, Li A, Zhang Y, Li J, Du H (2015). Lentiviral vectors mediate long-term and high efficiency transgene expression in HEK 293T cells. Int J Med Sci.

[27] Huang J, Chen G, Wang J, Liu S, Su J (2022). Platycodin D regulates high glucose-induced ferroptosis of HK-2 cells through glutathione peroxidase 4 (GPX4). Bioengineered.

[28] te Poele JA, van Kleef EM, van der Wal AF, Dewit LG, Stewart FA (2001). Radiation-induced glomerular thrombus formation and nephropathy are not prevented by the ADP receptor antagonist clopidogrel. Int J Radiat Oncol Biol Phys.

[29] Azzam P, Francis M, Youssef T, Mroueh M, Daher AA, Eid AA (2021). Crosstalk between SMPDL3b and NADPH oxidases mediates radiation-induced damage of renal podocytes. Front Med (Lausanne).

[30] Meng XM, Ren GL, Gao L, Yang Q, Li HD, Wu WF (2018). NADPH oxidase 4 promotes cisplatin-induced acute kidney injury via ROS-mediated programmed cell death and inflammation. Lab Invest.

[31] Yang Q, Ren GL, Wei B, Jin J, Huang XR, Shao W (2019). Conditional knockout of TGF-betaRII/Smad2 signals protects against acute renal injury by alleviating cell necroptosis, apoptosis and inflammation. Theranostics.

[32] Liu L, Xie D, Xie H, Huang W, Zhang J, Jin W (2019). ARHGAP10 inhibits the proliferation and metastasis of CRC cells via blocking the activity of RhoA/AKT signaling pathway. Onco Targets Ther.

[33] Liu J, Zhang C, Wu H, Sun XX, Li Y, Huang S (2020). Parkin ubiquitinates phosphoglycerate dehydrogenase to suppress serine synthesis and tumor progression. J Clin Invest.

[34] Jiang M, Wei Q, Wang J, Du Q, Yu J, Zhang L (2006). Regulation of PUMA-alpha by p53 in cisplatin-induced renal cell apoptosis. Oncogene.

[35] Liu J, Yuan Q, Ling X, Tan Q, Liang H, Chen J (2017). PARP-1 may be involved in hydroquinone-induced apoptosis by poly ADP-ribosylation of ZO-2. Mol Med Rep.

[36] Huang YF, Niu WB, Hu R, Wang LJ, Huang ZY, Ni SH (2018). FIBP knockdown attenuates growth and enhances chemotherapy in colorectal cancer via regulating GSK3beta-related pathways. Oncogenesis.

[37] Wang XS, Sheng Z, Ruan YB, Guang Y, Yang ML (2005). CpG oligodeoxynucleotides inhibit tumor growth and reverse the immunosuppression caused by the therapy with 5-fluorouracil in murine hepatoma. World J Gastroenterol.

[38] Hamad R, Jayakumar C, Ranganathan P, Mohamed R, El-Hamamy MM, Dessouki AA (2015). Honey feeding protects kidney against cisplatin nephrotoxicity through suppression of inflammation. Clin Exp Pharmacol Physiol.

[39] Chen D, Zeng R, Teng G, Cai C, Pan T, Tu H (2021). Menstrual blood-derived mesenchymal stem cells attenuate inflammation and improve the mortality of acute liver failure combining with A2AR agonist in mice. J Gastroenterol Hepatol.

[40] Mai B, Han L, Zhong J, Shu J, Cao Z, Fang J (2022). Rhoifolin alleviates alcoholic liver disease in vivo and in vitro via inhibition of the TLR4/NF-kappaB signaling pathway. Front Pharmacol.

[41] Heale JT, Ball AR, Schmiesing JA, Kim JS, Kong X, Zhou S (2006). Condensin I interacts with the PARP-1-XRCC1 complex and functions in DNA single-strand break repair. Mol Cell.

[42] Keil C, Grobe T, Oei SL (2006). MNNG-induced cell death is controlled by interactions between PARP-1, poly(ADP-ribose) glycohydrolase, and XRCC1. J Biol Chem.

[43] El-Khamisy SF, Masutani M, Suzuki H, Caldecott KW (2003). A requirement for PARP-1 for the assembly or stability of XRCC1 nuclear foci at sites of oxidative DNA damage. Nucl Acids Res.

[44] Dawson LA, Kavanagh BD, Paulino AC, Das SK, Miften M, Li XA (2010). Radiation-associated kidney injury. Int J Radiat Oncol Biol Phys.

[45] Zhang C, Zhao H, Li BL, Fu G, Liu H, Cai JM (2018). CpG-oligodeoxynucleotides may be effective for preventing ionizing radiation induced pulmonary fibrosis. Toxicol Lett.

[46] Chen J, Tian X, Mei Z, Wang Y, Yao Y, Zhang S (2016). The effect of the TLR9 ligand CpG-oligodeoxynucleotide on the protective immune response to radiation-induced lung fibrosis in mice. Mol Immunol.

[47] Zhang C, Ni J, Gao F, Sun D, Zhou C, Cheng Y (2011). The mechanism for the ameliorative effect of CpG-oligodeoxynucleotides on bone marrow hemopoiesis radiation injury. Basic Clin Pharmacol Toxicol.

[48] Zhang C, Zheng M, Zhu XH, Li S, Ni J, Li BL (2014). Protective effect of CpG-oligodeoxynucleotides against low- and high-LET irradiation. Cell Physiol Biochem.

[49] Ignacio RM, Kim CS, Kim YD, Lee HM, Qi XF, Kim SK (2015). Therapeutic effect of active hexose-correlated compound (AHCC) combined with CpG-ODN (oligodeoxynucleotide) in B16 melanoma murine model. Cytokine.

[50] Li X, Xu G, Qiao T, Yuan S, Zhuang X (2016). Effects of CpG oligodeoxynucleotide 1826 on acute radiation-induced lung injury in mice. Biol Res.

[51] Abdel-Magied N, Shedid SM, Ahmed AG (2019). Mitigating effect of biotin against irradiation-induced cerebral cortical and hippocampal damage in the rat brain tissue. Environ Sci Pollut Res Int.

[52] Said RS, El-Demerdash E, Nada AS, Kamal MM (2016). Resveratrol inhibits inflammatory signaling implicated in ionizing radiation-induced premature ovarian failure through antagonistic crosstalk between silencing information regulator 1 (SIRT1) and poly(ADP-ribose) polymerase 1 (PARP-1). Biochem Pharmacol.

[53] Yu JT, Hu XW, Yang Q, Shan RR, Zhang Y, Dong ZH (2022). Insulin-like growth factor binding protein 7 promotes acute kidney injury by alleviating poly ADP ribose polymerase 1 degradation. Kidney Int.

[54] Shin HJ, Kwon HK, Lee JH, Gui X, Achek A, Kim JH (2015). Doxorubicin-induced necrosis is mediated by poly-(ADP-ribose) polymerase 1 (PARP1) but is independent of p53. Sci Rep.

[55] Singh MP, Chauhan AK, Kang SC (2018). Morin hydrate ameliorates cisplatin-induced ER stress, inflammation and autophagy in HEK-293 cells and mice kidney via PARP-1 regulation. Int Immunopharmacol.

[56] Kim J, Long KE, Tang K, Padanilam BJ (2012). Poly(ADP-ribose) polymerase 1 activation is required for cisplatin nephrotoxicity. Kidney Int.

[57] Hanzlikova H, Gittens W, Krejcikova K, Zeng Z, Caldecott KW (2017). Overlapping roles for PARP1 and PARP2 in the recruitment of endogenous XRCC1 and PNKP into oxidized chromatin. Nucl Acids Res.

[58] Polo LM, Xu Y, Hornyak P, Garces F, Zeng Z, Hailstone R (2019). Efficient single-strand break repair requires binding to both poly(ADP-Ribose) and DNA by the central BRCT domain of XRCC1. Cell Rep.

[59] Yang CC, Yip HK, Chen KH, Sun CK, Chen YT, Chai HT (2017). Impact of impaired cardiac function on the progression of chronic kidney disease—role of pharmacomodulation of valsartan. Am J Transl Res.

[60] Wolf N, Pendergrass W, Singh N, Swisshelm K, Schwartz J (2008). Radiation cataracts: mechanisms involved in their long delayed occurrence but then rapid progression. Mol Vis.

