# Supplementary figures and images for: Cytosine–phosphate–guanine oligodeoxynucleotides alleviate radiation-induced kidney injury in cervical cancer by inhibiting DNA damage and oxidative stress through blockade of PARP1/XRCC1 axis
Source: J Transl Med. 2023 Sep 29;21:679. doi: 10.1186/s12967-023-04548-y (PMC10541701; doi:10.1186/s12967-023-04548-y)

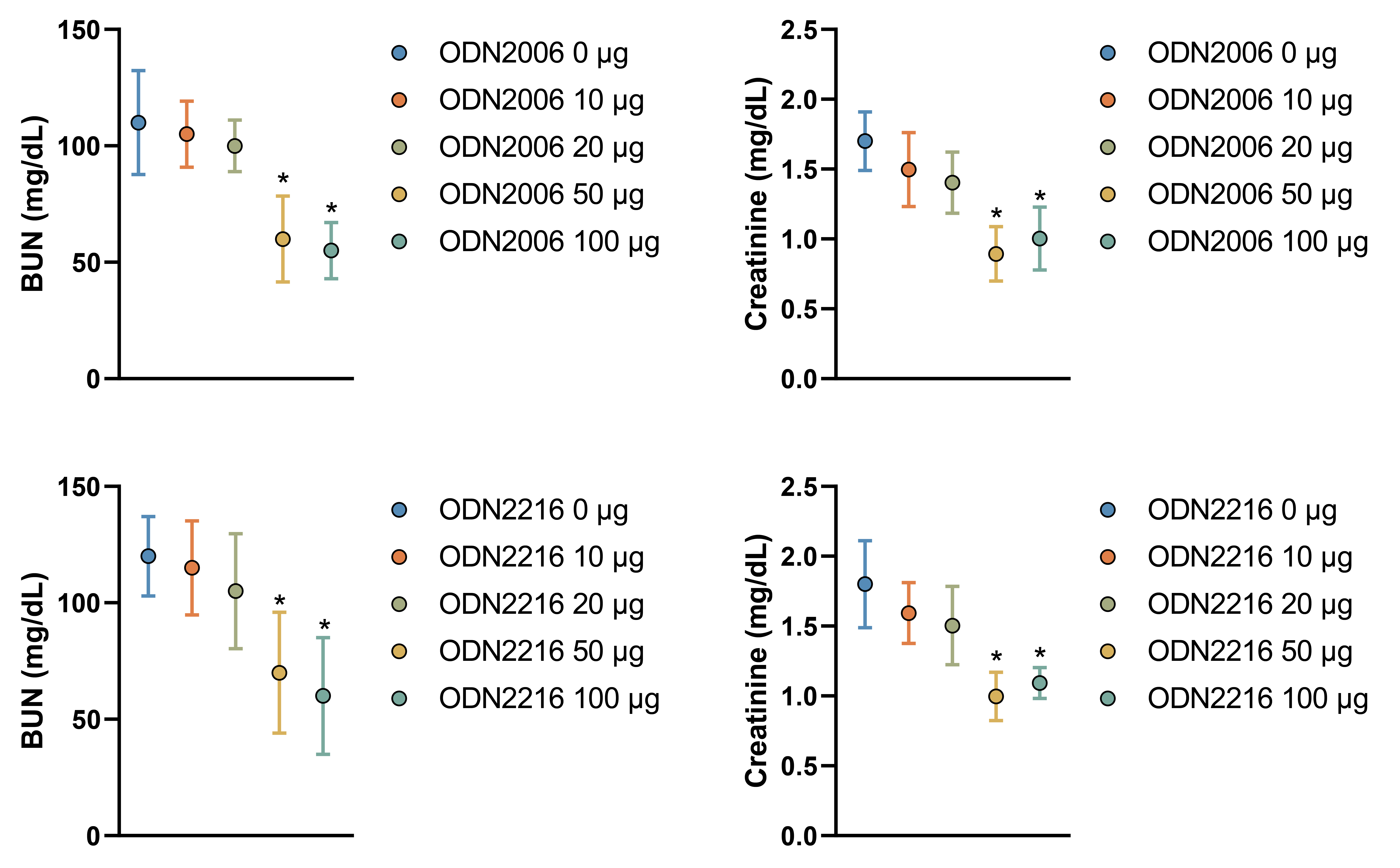

Supplement: Supplementary file 1 — Additional file 1: Fig. S1. Measurement of BUN and Creatinine Levels in Mouse Serum under Different Concentrations of CpG-ODNs Treatment Using an Automated Biochemical Analyzer. Note: Intraperitoneal injection in mice was used to validate the optimal concentration of ODN2006 and ODN2216. BUN and Creatinine levels in serum were measured at concentrations ranging from 0 to 100 μg. Significant differences were observed at concentrations of 50 μg and 100 μg. [file 12967_2023_4548_MOESM1_ESM.jpg]

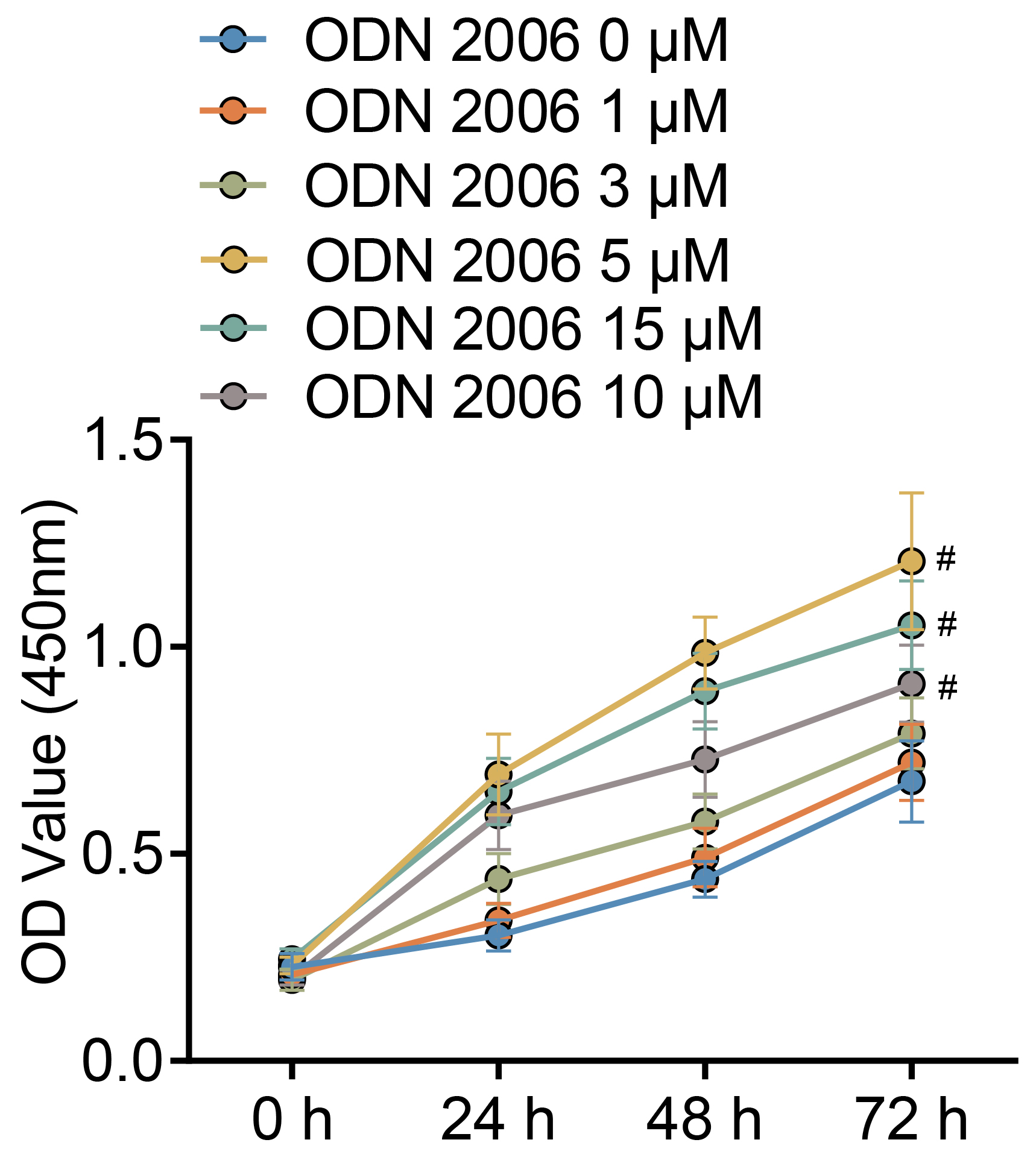

Supplement: Supplementary file 2 — Additional file 2: Fig. S2. Measurement of HK-2 cell viability under different concentrations of CpG-ODNs treatment and radiation-induced damage using CCK-8 assay. [file 12967_2023_4548_MOESM2_ESM.jpg]
