# Supplementary material for: Cytosine–phosphate–guanine oligodeoxynucleotides alleviate radiation-induced kidney injury in cervical cancer by inhibiting DNA damage and oxidative stress through blockade of PARP1/XRCC1 axis
Source: J Transl Med. 2023 Sep 29;21:679. doi: 10.1186/s12967-023-04548-y (PMC10541701; doi:10.1186/s12967-023-04548-y)
Supplement: Supplementary file 3 — Additional file 3: Table S1. shRNA sequences. Table S2. The treatment conditions of the different groups of mice. Table S3. Primer sequences for RT-qPCR. [file 12967_2023_4548_MOESM3_ESM.docx]

**Table S1** shRNA sequences

| Name | shRNA sequence |
| --- | --- |
| shRNA-NC | Sense:5’-CACCCAACAAGATGAAGAGCACCAACGAATTGGTGCTCTTCATCTTGTTG-3’ |
|  | Anti-sense:5’-AAAAGTTGTTCTACTTCTCGTGGTTTTCGAACCACGAGAAGTAGAACAAC-3’ |
| shRNA-PARP1-1 | Sense:5’-CACCGCAAAGGCCAGGATGGAATTGCGAACAATTCCATCCTGGCCTTTGC-3’ |
|  | Anti-sense:5’-AAAAGCAAAGGCCAGGATGGAATTGTTCGCAATTCCATCCTGGCCTTTGC-3’ |
| shRNA-PARP1-2 | Sense:5’-CACCGCCAAGTCCAACAGAAGTACGCGAACGTACTTCTGTTGGACTTGGC-3’ |
|  | Anti-sense:5’-AAAAGCCAAGTCCAACAGAAGTACGTTCGCGTACTTCTGTTGGACTTGGC-3’ |
| shRNA-XRCC1-1 | Sense:5’-CACCGGGAATGATGGCTCAGCTTTCCGAAGAAAGCTGAGCCATCATTCCC-3’ |
|  | Anti-sense:5’-AAAAGGGAATGATGGCTCAGCTTTCTTCGGAAAGCTGAGCCATCATTCCC-3’ |
| shRNA-XRCC1-2 | Sense:5’-CACCGCAAGACTATGAGGTCCTTCTCGAAAGAAGGACCTCATAGTCTTGC-3’ |
|  | Anti-sense:5’-AAAAGCAAGACTATGAGGTCCTTCTTTCGAGAAGGACCTCATAGTCTTGC-3’ |

Note: NC, negative control; PARP1, poly(ADP-ribose) polymerase 1; XRCC1, X-ray repair cross complementing 1.

**Table S2** The treatment conditions of the different groups of mice

| Gene | Sequence (5'-3') |
| --- | --- |
| Normal | Normal mice not subjected to X-ray irradiation treatment |
| Normal+ODN2006 | Normal mice injected intraperitoneally with ODN2006 |
| Normal+ODN2216 | Normal mice injected intraperitoneally with ODN2216 |
| RKI+Saline | RKI model mice injected with saline solution. |
| RKI+ODN2006 | RKI model mice injected intraperitoneally with ODN2006. |
| RKI+ODN2216 | RKI model mice injected intraperitoneally with ODN2216 |
| RKI+Saline+oe-NC | RKI model mice injected intraperitoneally with saline solution and oe-NC lentivirus |
| RKI+ODN2006+oe-NC | RKI model mice injected intraperitoneally with ODN2006 and oe-NC lentivirus |
| RKI+ODN2006+oe-PARP1 | RKI model mice injected intraperitoneally with ODN2006 and oe-PARP1 lentivirus |
| RKI+ODN2006+oe-XRCC1 | RKI model mice injected intraperitoneally with ODN2006 and oe-XRCC1 lentivirus |

**Table S3** Primer sequences for RT-qPCR

| Gene | Sequence (5'-3') |
| --- | --- |
| PARP1 (human) | Forward: 5'-CGGAGTCTTCGGATAAGCTCT-3'  Reverse: 5'-TTTCCATCAAACATGGGCGAC-3' |
| PARP1 (mouse) | Forward: 5'-GGCAAGCACAGTGTCAAAGG-3'  Reverse: 5'-TGTCGTTGACACCAGATGGG-3' |
| GAPDH (mouse) | Forward: 5'-CATGGCCTTCCGTGTTCCTA-3'  Reverse: 5'- CACAGGAGACAACCTGGTCC-3' |
| GAPDH (human) | Forward: 5'-AAAGCCTGCCGGTGACTAAC-3'  Reverse: 5'-TTCCCGTTCTCAGCCTTGAC-3' |

Note: RT-qPCR, reverse transcription-quantitative polymerase chain reaction; PARP1, poly(ADP-ribose) polymerase 1; GAPDH, glyceraldehyde-3-phosphate dehydrogenase.
